# Down the brain drain: a rapid review exploring physician emigration from West Africa

**DOI:** 10.1186/s41256-023-00307-0

**Published:** 2023-06-27

**Authors:** Tega Ebeye, HaEun Lee

**Affiliations:** 1 Temerty Faculty of Medicine, University of Toronto, Toronto, Canada; 2 Institute of Health Policy, Management and Evaluation, University of Toronto, Toronto, Canada; 3 Center for Global Health Equity, University of Michigan, Ann Arbor, USA

**Keywords:** Physician emigration, West Africa, Brain drain, Health workforce, Globalization

## Abstract

**Background:**

The emigration of physicians from low- and middle-income countries (LMICs) to high-income countries (HICs), colloquially referred to as the “brain drain”, has been a topic of discussion in global health spheres for years. With the call to decolonize global health in mind, and considering that West Africa, as a region, is a main source of physicians emigrating to HICs, this rapid review aims to synthesize the reasons for, and implications of, the brain drain, as well as recommendations to mitigate physician emigration from West African countries to HICs.

**Methods:**

A literature search was conducted on PubMed, EMBASE and The Cochrane Library. Main inclusion criteria were the inclusion of West African trained physicians' perspectives, the reasons and implications of physician emigration, and recommendations for management. Data on the study design, reasons for the brain drain, implications of brain drain, and proposed solutions to manage physician emigration were extracted using a structured template. The Hawker Tool was used as a risk of bias assessment tool to evaluate the included articles.

**Results:**

A total of 17 articles were included in the final review. Reasons for physician emigration include poor working conditions and remuneration, limited career opportunities, low standards of living, and sociopolitical unrest. Implications of physician emigration include exacerbation of low physician to population ratios, and weakened healthcare systems. Recommendations include development of international policies that limit HICs’ recruitment from LMICs, avenues for HICs to compensate LMICs, collaborations investing in mutual medical education, and incorporation of virtual or short-term consultation services for physicians working in HICs to provide care for patients in LMICs.

**Conclusions:**

The medical brain drain is a global health equity issue requiring the collaboration of LMICs and HICs in implementing possible solutions. Future studies should examine policies and innovative methods to involve both HICs and LMICs to manage the brain drain.

## Background

Physician emigration from low- and middle-income countries (LMICs) to high-income countries (HICs), colloquially referred to as the “*brain drain*”, has been a topic of discussion in global health spheres for years. Sub-Saharan Africa—predominantly West Africa—serves as a major source region of physicians emigrating to high-income countries. Of the international medical graduates practicing in the US from sub-Saharan African countries, 44.5% are from Nigeria; with the World Health Organization (WHO) sponsored *Global Health Work Alliance* estimating that 1 in 4 physicians will leave Africa to pursue jobs abroad [1, 2]. Between 2005 and 2015, there was over a 70% increase in the number of Africa-trained physicians who subsequently entered the US workforce [1, 3].

Furthermore, most “supplier countries'' already have fragile health systems that are not insulated from outside factors such as economic factors, political factors, conflict, and unrest [4]. Thus, it is apparent that most countries experiencing a crisis in human resources for health (HRH) are in sub-Saharan Africa, many of them conflict-afflicted and struggling with the concurrent flight of human capital, mismatches between skills and service needs, breakdown of pre-service training, and lack of human resource data [4].

Given the importance of West Africa in the brain drain phenomenon, with the ongoing and underexplored emigration of physicians specifically from West African countries, this rapid review aims to explore the volume and quality of existing literature on physician emigration from West African countries, reasons for emigration, implications of emigration, and recommendations to mitigate the mass exodus of physicians from West African countries. In addition, with the call to decolonize global health, it is pertinent that the discourse of the brain drain—particular to sub-Saharan Africa—is ongoing; with emphasis on highlighting voices from within the continent in a capacity building, asset-based community development (ABCD) approach which intentionally counteracts the deficit-oriented mentalities that reinforce colonial power differentials [5]. Thus, ultimately serving as initial steps to advance the movement of decolonization of global health from reflection to action [6].

The rationale behind conducting a rapid review hinges on the need for a quick, concise, summary of recommendations to address the brain drain, particularly of physicians from West Africa, in order to direct further policies and research, specifically regarding implementation science. This is especially important at such a pivotal time for global health, with increasing globalization amidst emerging threats to the stability of health systems.

## Methods

### Protocol and search strategy

Using the WHO framework for rapid reviews [7] as a guide, we conducted a literature search of three databases—PubMed, EMBASE and The Cochrane Library. The keywords and Boolean operators “*physician OR doctor*”, AND “*emigration*”, AND “*West Africa*” were used to conduct the search throughout the various databases. The search was only filtered by date of publication as the review sought to include articles written within the last twenty years (from 2001 to 2021) to capture current voices on this issue.

### Study selection and eligibility criteria

Titles and abstracts were further screened by two independent researchers (TE and HD). Inclusion criteria were articles that: (1) captured West African trained physicians’ perspectives about emigration, (2) explored implications of physician emigration from West African countries, and (3) included recommendations for managing the emigration of West African trained physicians. Studies that were older than 20 years old, written in a language other than English, did not include physicians from West Africa, or did not pertain to the topic of physician emigration were excluded. Once the suitability of the studies had been assessed using titles and abstracts, the full texts were further assessed for their suitability by two independent researchers (TE and HD).

### Data extraction

The selected studies’ full texts were sent for data extraction. Data extraction was conducted via a pre-established template in Covidence (*Covidence systematic review software, Veritas Health Innovation, Melbourne, Australia*) a web-based collaboration software that streamlines the process of reviews. Extracted data included study settings, study designs, reasons for, implications of the brain drain, and potential solutions. The data extraction was independently carried out by two researchers (TE and HD), with the lead researcher confirming consensus.

### Method of synthesis

Following data extraction, the strategy for data synthesis applied a narrative description of the selected studies in the form of tables and text. Given the qualitative and narrative nature of the studies, the approach involved summarizing key points from each included study that highlighted the reasons for, and implications of physician emigration from West Africa, as well as recommendations to manage this ongoing exodus.

### Risk of bias

A risk of bias assessment was conducted to assess the quality of included studies. The *Hawker Tool* was used as a guideline to further direct exclusion of poor-quality studies—which, for this review, is defined as scoring 18 and below on the *Hawker Tool* [8]. The tool contains nine questions on clarity and appropriateness of articles’ structure, sampling methods, data analysis, ethics, and transferability, each of which can be answered ‘good’, ‘fair’, ‘poor’ or ‘very poor’ and scored from 1 point (very poor) to 4 points (good); producing a score for each study of a minimum of 9 points and a maximum of 36 points [9]. The nine questions included in the tool are graded per the prescribed checklist of the *Hawker Tool* [8]*.*

## Results

### Search results

**Fig. 1:**
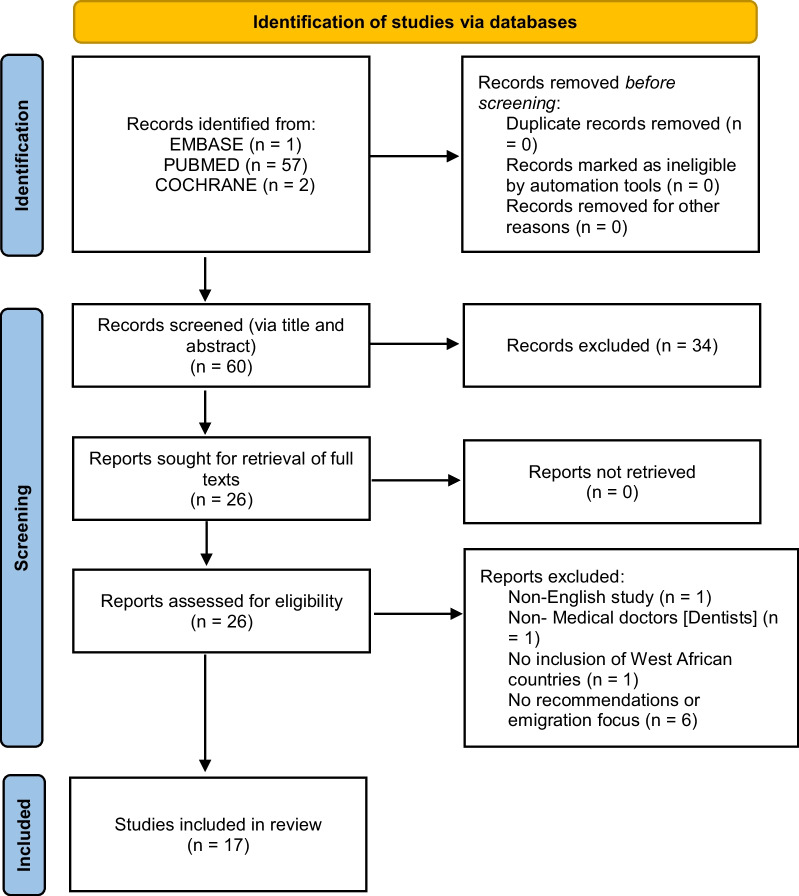
Preferred Reporting Items for Systematic Reviews and Meta-Analysis (PRISMA) diagram for the process of the article extraction. *Source*: Flowchart adapted from the available template provided by PRISMA at http://prisma-statement.org/

A total of 60 articles were retrieved form the three databases. After titles and abstracts were screened, 26 articles remained. Of these, nine articles were further removed after full-text review, with 17 articles remaining for final inclusion. See Fig. 1 for the Preferred Reporting Items for Systematic Reviews and Meta-Analysis (PRISMA) flow chart [10] illustrating the study selection process.

In alignment with the purpose of this rapid review, we summarized and reported the West African country included in the study, the study design, the reasons for emigration, the implications of the brain drain, and recommendations to mitigate. The extraction summary table is presented in Table 1*.*

**Table 1:** Summary of reasons for, implications of, and recommendations to mitigate the medical brain drain

References	West African countries included	Study design	Reasons for emigration	Implications of the brain drain	Recommendations
Astor et al. [12]	Nigeria	Cross-sectional, qualitative study	Low pay/income Limited access to enhanced technology Limited medical jobs Limited opportunities to work in academic environment Less prestige associated with being a physician compared to being a physician in HICs Concerns regarding safety Limited prospect for children	Insufficient medical expertise within supplier country Insufficient number of physicians for population Improvements in medical knowledge and education via increased number of medical schools and increased commercialization of medical schools Promote international collaboration for healthcare research and development Effects are felt worse in rural areas of countries as well as more by public sector than private	Increase physician income Improve physician-working conditions Regulate physician migration through government migration control Require physicians to compensate supplier country if they chose employment abroad Require recipient countries to compensate supplier country Require medical graduates to work in home country for a set amount of time after graduation Increase formal partnership between medical schools of supplier and recipient countries with explicit conditions of return Improve government-level monitoring of physician flow
Eastwood et al. [13]	Nigeria and Ghana	Commentary	Limited postgraduate training opportunities Underfunding of health-service facilities Poor remuneration Poor governance and health-service management Civil unrest and personal security Considerably ‘easy’ transition to other English-speaking countries	Shortage of healthcare personnel, especially in rural areas of supplier country	Train more doctors in the UK Increase aid and technical assistance via the Department for International Development (DfID) to assist in recruitment and retention of health care professionals in supplier countries (especially rural areas) Restrict duration of training for health workers from supplier countries Reshape and strengthen supplier country incentive schemes for physicians Agreement between recipient countries and the WHO about standard for the *minimum* HCP training targets in developing countries
Eliason [2]	Ghana	Cross-sectional, qualitative study	Limited opportunities for professional development Limited socioeconomic compensation High cost for medical education push trainees to emigrate to high-paying countries	N/A	Supplier countries like Ghana should explore funding mechanisms that are less burdensome to the medical trainees and their families
Hagopian et al. [14]	Nigeria and Ghana	Cross-sectional, qualitative study	Poor working conditions Poor renumeration Limited training and research opportunities Limited post-graduate training opportunities Political and economic conditions of the country Poor working environment and infrastructures Regular labor strikes	Rural and poor communities have limited access to health services Inadequate physician leaders to advance health system Limited ability for the health sector to develop and expand Depletes an important element of the middle class made up by African physicians which leads to an increase of the proportion of the population living in poverty	Improve physician incentives Create barriers for emigration Recoup financial investment losses from emigrating physicians Expand scope of other healthcare providers' work Receive compensation from receiving countries
Ike [15]	Nigeria	Commentary	These are categorized into three: (1) Global factors: Pressure on health providers in recipient countries who become desperate for more workers especially in rural areas Profit by recruitment agencies Professionals (2) The push factors: Poor working conditions Poor wages Lower standards of living Underutilization of qualified personnel Political instability Declining educational systems Mediocrity and discrimination Limited civil liberties Social insecurity (3) Related determinants: Have family members living abroad Better opportunities for children Ability to send remittances home	Health of population affected	Utilize skills of the other health professionals Enhance remuneration to attract Nigerian health professionals to remain and/or return to the supplier country Collect better data on physician emigration Increase national budget for education and healthcare Invest in infrastructure and rehabilitation of healthcare systems Adapt “virtual participation,” encouraging highly skilled expatriates to contribute their experiences to the development of supplier country without necessarily physically relocating Improve governance, accountability, and transparency within government
Jenkins et al. [16]	Nigeria, Ghana, Congo, Cameroon, Liberia, Senegal, and Sierra Leone	Cross-sectional, qualitative study	Low salaries Poor occupational safety Inadequacy of facilities and supply of medicines Lack of post graduate training and continuing professional development Lack of a multidisciplinary approach Poor treatment conditions for patients Limited mentors/supervisor for advanced practice	Loss of health system capacity to deliver health care equitably	Increase educational opportunities such as professional development, research opportunities, and scholarships, Improve working environment such as flexible working hours, better facilities and equipment Improve incentives such as provision of housing, transportation, and childcare Improve security Expand other healthcare provider training and roles
Karan et al. [11]	Nigeria	Commentary	Limited training opportunities Low salaries Political instability and corruption Poor quality of facilities and equipment Concern for family and children’s future	Suboptimal health systems functioning and quality of care	HICs reduce both passive and active recruitment of physicians from LMICs The US and other recipient countries should work to alleviate the physician shortage in supplier country by providing educational loan forgiveness to clinicians working in underserved areas in the US Recipient country pay fee to supplier countries in order to recruit physicians from these countries Expand roles and training for less skilled healthcare workers Customize medical curricula in source countries to be more locally relevant. This could increase social prestige and compensation when physicians remain local Increase opportunities for career advancement
Loh et al. [20]	Ghana	Cross-sectional, qualitative study	Poor income Better professional prospects and higher standards of living abroad The gap in investigation of the factors related to health care delivery and financing that could drive emigration Public health care delivery and financing may increase physician emigration when compared to private	Drains skilled personnel from already weak health systems Reduces the success of existing primary care and public health activities	Encouraging private health-care delivery and financing may decrease physician emigration. However, may possibly affect the availability and quality of universal health-care coverage
Mullan 2005 [24]	Nigeria and Ghana	Cross-sectional, quantitative study	Limited medical-training positions Limited opportunity for medical employment	Supplier countries lose important healthcare capabilities Increased health inequity, health disparities such as human immunodeficiency virus (HIV) infection and the acquired immunodeficiency syndrome (AIDS) related deaths	Increase investments by recipient nations in own medical education Assist LMICs to retain physicians and focus training on national needs rather than on the international physician market
Mullan 2007 [21]	Ghana	Commentary	Poor pay even though the salaries of Ghanaian doctors are better than those in many African countries	Maternal and infant mortality rates (in Ghana) are more than 10 times those of high-income countries	Supplier country train more physicians in the hopes to retain more Receiving country train more physicians for themselves Receiving countries send their physicians to work in supplier countries Increase pay, provide loans and subsidized housing for physicians increases Expand in-country medical residency programs Expand education and training for community health nurses, technical officers, and medical assistants to substitute for doctors in shortage areas
Nwadiuko et al. [22]	Nigeria	Cross-sectional, quantitative study	Insufficient physical security Lack of economic security	Supplier country’s health systems suffer from the gap left by emigrated physicians Increased HIV mortality related to physician emigration	Outside organizations can partner with emigrated physicians to advise individual and network level contributions For emigrated physicians preparing to permanently relocate back to their countries of origin, the private sector might offer an attractive option, although governmental support remains necessary for their successful integration
Okeke [23]	Ghana	Cross-sectional, qualitative study	Low wages	Worsened doctor-to-population ratios in LMICs	Increase the salaries of health professionals in LMICs Compensate doctors for overtime work
Opoku and Apenteng [17]	Ghana	Cross-sectional, quantitative study	Low salary Job dissatisfaction Poor working conditions and living conditions Limited research and working opportunities	Magnify the already existing shortages of healthcare providers Loss of investment for supplier nations countries	Advance data tracking physician emigration Improve remuneration Enhance career development and continuing education opportunities Improve resource availability and working conditions HICs decrease their dependence on international graduates by increasing their own capacity of healthcare workforce HICs compensate LMICs for their human resource loss and/or establish bilateral policies to decrease impact of physician emigration from LMICs to HICs
Ossai et al. [18]	Nigeria	Cross-sectional, quantitative study	Limited spaces for internships, residencies, and formal employment after training Limited options for specialization in career Nation-wise socioeconomic or political unrest Limited opportunities family and children in supplier country Limited infrastructure, facility, and equipment	Critical shortage of health workers Poor quality health services in supplier countries	More rural placements to increase rural interest Government-level compensation and appreciation for physicians remaining in LMICs Physicians who are trained abroad can also be brought back to advance supplier country’s specialist training
Udonwa [19]	Nigeria	Commentary	Limited facilities especially in rural areas Limited opportunities for medical specialities Limited economic inequalities that exacerbates salaries between HICs and LMICs Corrupt leadership, political upheaval, and/or civil unrest	Widening difference of healthcare outcomes between rural and urban areas	Mobilize physicians who emigrated to HICs and achieved professional success to undertake short-term consultancies in their countries of origin Train more staff to reduce human capital impact Refrain from erecting legal barriers to the emigration of educated professionals which will only encourage illegal emigration; instead, enact necessary economic reforms that make staying at home rewarding for educated Nigerians Good leadership and policy planning Good governance at the national and international levels Improved security for peoples' lives and property Investment in more research and policy about the causes of the drain and in educating policy makers about the causes Improved wages according to physician qualifications Offering better quality education and expanding educational infrastructure Implement tax to physicians who are wishing to emigrate Government level agreement between LMICs and HICs to discourage physician emigration Contract medical students to refund their education fee if they leave the country before a minimum service period (return of service)
Woodward [3]	Sierra Leone	Cross-sectional, qualitative study	Sociopolitical unrest Frequent disease outbreaks (like Ebola) Increased workload	Fragile healthcare system becoming more vulnerable and overburdened	Development of postgraduate medical education in low-income and crisis-affected countries
Wright et al. [25]	Non-specific mention of English-speaking commonwealth countries (which includes multiple West African countries)	Commentary	Low standard of living	Depletion of human resources of health in LMICs	Improve pay Better and safer working conditions Fewer patient caseload Address political instability and personal safety Increase domestic supply of physicians Develop compensatory schemes from receiving countries Policy initiatives to stop recruiting from LMICs

### Study characteristics

Of the 17 articles included, 7 (41.2%) used a primarily qualitative studies, 6 (35.3%) were commentaries, and 4 (23.5%) were quantitative studies. Six studies were focused on Nigeria, five on Ghana, three on both Nigeria and Ghana, one on Sierra Leone, and the rest on a combination of Nigeria, Ghana, Sierra Leone, Liberia, Senegal, Congo, Cameroon, and/or non-specific commonwealth countries in West Africa.

### Reasons for physician emigration

Reasons for physician emigration are often described as “push and pull factors”, in which push factors are shortcomings that lead to emigration of physicians from LMICs, while the characteristics in HICs that compensate these limitations are the pull factors for physicians to emigrate to these countries [3]. The push factors as reasons for emigration were summarized to include poor working conditions, low standards of living, limited career opportunities, and sociopolitical unrests. Poor working conditions includes limited infrastructures, equipment, and staff [11–19]. These poor working conditions were exacerbated in recent disease outbreaks such as Ebola [3] and the COVID-19 pandemic; which further caused increased workloads with physicians providing suboptimal quality of care. Subsequently, poor renumeration and benefits were included under poor working conditions [2, 11–20, 22]. Renumeration was often insufficient for physicians to pay off their student loans and provide for their families [2, 18]. Additionally, overtime pay and workers’ compensation (related to work injuries) were close to non-existent. These affected standards of living, which then served as an additional push factor driving emigration to countries with better prospects for physicians and their families [11, 12, 15, 18–20].

Limited career opportunities such as residency programs and research opportunities, as well as a general lack of mentors to learn from were significant push factors [2, 11–14, 16–20, 24]. Furthermore, sociopolitical unrest affecting personal and family safety were also emphasized as reasons for emigration [3, 11–16, 18, 19, 22]. This was occasionally tied in with government level corruption, lack of transparency and accountability, and limited prioritization of healthcare systems which also contributed to decisions to emigrate [11, 19].

### Implications of physician emigration

The implications of physician emigration from West Africa affect healthcare systems and the population concurrently. With physicians leaving their home countries, the physician to population ratio increases [12–15, 17–19, 23]. The limited number of physicians disproportionately affects the poor, especially those living in rural communities; [12–14, 19] as, for improved pay and working conditions, the remaining physicians often transition to private practice in urban centers which decreases access to care for poor and rural communities [20]. Furthermore, vulnerable populations—such as pregnant women, children under five years old and people living with HIV/AIDS—tend to suffer greatly from this insufficient, suboptimal care created and exacerbated by the brain drain [21, 22, 24].

Physician emigration further increases the workload of the remaining physicians in West Africa, thus, accelerating burnout, which in turn, decreases quality of care [11, 15, 18]. Low physician census in these countries also means less physicians in leadership and administrative roles to help advance medicine, health policy, and thus, health systems [14, 20]. Ultimately, the brain drain further weakens health systems in West Africa [3, 11, 14, 16, 17, 20, 22, 24, 25].

### Recommendations to mitigate physician emigration

First, seeing as push factors revolve around remuneration and working conditions, West African countries need to arrive at policy decisions that improve these aspects if they intend to improve physician retention. These can include compensation for overtime work, subsidizing housing and transportation fees (for financial and safety purposes) as well as a general increase in pay that matches inflation rates [12, 14–19, 21, 23, 25]. Furthermore, these changes need to prioritize solutions addressing the national level issues of corruption, governance, and security [15, 19, 25].

In addition, role expansion of allied healthcare workers has the potential to lighten physician workload and compensate for low physician census [14–16, 21]. Additionally, suggestions around investment in educational opportunities such as scholarships for research, and professional development reoccurred as a theme to improve retention [3, 16, 17, 19, 21, 24]. It was also posed that physicians educated with government funding can be expected to provide a return of service for certain number of years after graduation, ensuring that physician training is customized to meet LMICs needs rather than the international physician market [11, 24].

Somewhat controversially, national policies to restrict and regulate physician emigration were posed, with the potential for the imposition of “emigration fees” levied against physicians who decide to pursue employment overseas, [12, 14, 19] as well as a limit on the number of years physicians can train overseas [12, 13]. These will be coupled with specific targets to bring back physicians who have trained overseas; [15, 18, 22] with a potential substitute to relocating being short term consultations or virtual participation by physicians working in HICs, enabling them to remotely alleviate the burden of care without physically relocating [19, 20].

International policies which limit receiving countries’ ability to aggressively recruit physicians from supplier countries and require receiving countries to compensate supplier countries for recruited physicians were posed as potential solutions to the brain drain [11, 12, 14, 17, 19, 25]. Furthermore, the development of international partnerships where HICs train physicians from LMICs and/or send their physicians to LMICs, may address the equity issues arising from LMICs spending their limited resources to train physicians just for them emigrate [11, 12, 21]. Overall, there is still room for improvement on quality of data on physician emigration patterns, especially if solutions are to be specific and effective [12, 15, 17].

### Risk of bias assessment

Regarding the included studies’ risk of bias, none of the articles scored below the ascribed threshold of 18 on the risk of bias assessment using the *Hawker Tool* (see *Methods* above). Based on the proposed scale, 58.8% of the studies were of high quality (A; 30–36 points on the *Hawker Tool*) with 10 of the 17 studies meeting this threshold; 23.5% (4 studies) were of medium quality (B; 24–29 points); and 17.7% (3 studies) of low quality (C; 9–23 points) as described in Table 2. For this study, the risk of bias assessment was also conducted by two independent reviewers with the goal to further exclude any article scoring 18 or below. The included studies were given overall quality grades with the following definitions: high quality (A), 30–36 points, medium quality (B), 24–29 points [9]; and low quality (C), 9–23 points.

**Table 2:** Risk of bias assessment

Study ID	Abstract and title	Intro and aims	Method and data	Sampling	Data analysis	Ethics and bias	Results	Transferability or generalizability	Implications and usefulness	Total	Grade^a^
Astor et al. [12]	4	3	4	3	2	3	4	3	4	30	A
Eastwood et al. [13]	4	3	1	2	3	3	3	3	4	26	B
Eliason [2]	4	4	3	4	3	3	4	4	4	33	A
Hagopian et al. [14]	3	4	4	3	3	3	3	3	4	30	A
Ike [15]	3	3	1	1	3	2	3	2	3	21	C
Jenkins et al. [16]	4	4	4	2	4	3	4	3	4	32	A
Karan et al. [11]	4	2	2	1	3	3	4	3	4	26	B
Loh et al. [20]	4	4	3	2	3	3	3	3	4	29	B
Mullan 2005 [24]	4	4	4	2	3	3	4	3	3	30	A
Mullan 2007 [21]	1	2	1	2	2	2	3	3	4	20	C
Nwadiuko et al. [22]	4	4	4	4	4	2	4	4	4	34	A
Okeke [23]	4	4	3	3	4	4	4	4	4	34	A
Opoku and Apenteng [17]	4	4	4	4	4	2	4	4	4	34	A
Ossai et al. [18]	4	4	4	4	4	2	4	4	4	34	A
Udonwa [19]	3	3	1	1	2	3	2	2	3	20	C
Woodward [3]	4	4	4	4	4	2	4	3	3	32	A
Wright et al. [25]	4	3	1	1	2	3	3	3	4	24	B

^a^*A* high quality, 30–36 points, *B* medium quality, 24–29 points, *C* low quality, 9–23 points

## Discussion

The reasons for physician emigration from West African countries to HICs, as well as the implications of their emigration on the systems left behind are well established in the literature. The results of this rapid review further highlight that poor working conditions and remuneration, limited career opportunities, and sociopolitical unrest push physicians to seek better opportunities for themselves, their families, and their careers. It is estimated that LMICs, representing 48% of the global population, have 19% of all surgeons, 15% of anesthesiologists, and 29% of obstetricians [26]. Thus, the ongoing brain drain has a palpable impact on population health in West African countries, disproportionately affecting marginalized populations within those countries; [12–14, 16, 19] as, for improved pay and working conditions, the remaining physicians often transition to urban private practices, decreasing access to care for poor, rural communities [20]. Therefore, the brain drain further amplifies the health disparities within LMICs. Nevertheless, there is a gap in the literature exploring the challenges faced in host HICs after emigration, which may include racism, difficulty transferring licences and securing job promotions, as well as adapting to new norms and cultures.

Furthermore, the results of this review indicate that the literature lacks recommendations which provide ethical solutions to the brain drain that prioritize global health equity while refraining from infringing on physicians’ autonomy. The brain drain is highly inequitable in that HICs benefit from the physicians that LMICs have invested their scarce resources to train, which further contributes to the health disparities within LMICs [11–14]. In 2016, non-US born medical graduates comprised approximately one-fifth of practicing physicians in the United States, and the number is predicted to continue to increase [27]. However, of the approximately 154 medical schools in the United States, only 48 schools accept international applicants, with approximately 0.6% of the medical school matriculants being non-US citizens [28]. This issue is not novel to the US alone, as other studies have also cited Australia, Canada, and the United Kingdom as HICs playing significant roles in the physician brain drain [29–31]. In fact, it is estimated that the overall benefit to destination countries of recruiting trained doctors was largest for the United Kingdom at $2.7billion [31].

Regardless of the destination country at play, it is evident that a significant number of physicians practicing in HICs were trained outside those countries—in LMICs. Therefore, physician emigration needs to be addressed through a global health equity lens, with commitment from both HICs and LMICs to tackle this issue through mutually beneficial, power-balanced partnerships and processes, with the primary goal being equitable health outcomes [26]. This has been spearheaded by the WHO through the *Global strategy on Human Resources for Health: Workforce 2030* [32] which poses recommendations for *all* stakeholders and member states to align investment in human resources for health with the current and future needs of the population. [32]. The WHO further proposes addressing health worker shortages and improving distribution of health workers by using strengthened data on HRH for monitoring and ensuring accountability for the implementation of national and regional strategies [32].

Thus, mirroring the WHO recommendations in light of the results from this study, the following discussion themes are posed:

### 1. Education

The chronic under-investment in education and training of health workers in some countries, and the mismatch between education strategies in relation to health systems and population needs are exacerbating ongoing workforce shortages [32]. Therefore, HICs need to eliminate gatekeeping practices and actively pursue means to educate promising students from LMICs, thereby alleviating the burden of training from these countries, especially if these HICs are to supplement their workforce with physicians from LMICs. Furthermore, there should be sustainable initiatives to train and supplement the workforce of LMICs by using techniques such as role expansion of allied healthcare workers [14–16, 21], and appropriate utilization of telemedicine, which has become widely accepted due to limitations and lessons of the COVID-19 pandemic. Some suggestions around telemedicine involve virtual participation avenues for physicians living in HICs to provide services to their home countries [15, 19].

### 2. Employment

Opportunities to strengthen the skills and employment agenda within countries including re-skilling workers from declining sectors and industries of the economy (e.g., manufacturing, agriculture) to be redeployed in the health and social care sectors, maximizing the scope and utilization of allied health care workers, and assisting newly qualified students to enter the employment market, should be employed [14–16, 21, 30, 32]. Proper incentivization, especially with regards to deploying healthcare workers to underserved areas should be employed [32].The above-mentioned strategies should be done in tandem with strategies addressing the safety of living and working in regions healthcare workers are deployed to.

### **3. Surveillance strategies**

The importance of appropriate surveillance systems for planning capacity to improve HRH policy that quantify health workforce needs, demands and supply cannot be denied [32]. These strategies will aid proper monitoring and distribution of HRH with the goal of more equitable and efficient distribution of resources, which will ultimately point to areas for potential impact to the medical brain drain.

### 4. Funding, resource allocation and remuneration

LMICs need to prioritize and invest in their healthcare systems. These include investing in medical education, health facilities and equipment, expanding research and residency programs, and actively recruiting and securing physicians to be mentors, leaders, and administrators [16]. Physicians who have paid for their education out-of-pocket are shown to be more likely to emigrate to HICs [11]. Hence, expanding programs for government subsidized tuition fees with a projected return of services can serve as a promising solution. While it may be unreasonable to provide the same pay as physicians abroad, LMICs’ physicians still need improved renumeration and benefits This maybe in the forms of increased salary, overtime pay, subsidized housing and transportation. [11, 21, 25]. There are also deeply rooted national issues -like corruption, lack of accountability and transparency, and the ongoing unrest—that affect healthcare system stability and need to be addressed in order to aid retention [12, 16, 25]. Moreover, at the end of the day, physicians should not be faced with the ethical dilemma of choosing between what is best for their wellbeing, and their calling to meet the needs of their countryman [11, 19, 25]. Therefore, it is safe to infer that collaboration between the governments of LMICs and HICs, as well as their health system leaders, is needed to foster the structures that enable physicians to find a semblance of balance between wellbeing and calling.

By design, there are limitations to this rapid review. First, the search did not include synonyms and Medical Subject Headings (MeSH) as it was directed at extracting and summarizing information in a timely manner. Secondly, exclusion of articles written in languages other than English, as well as the inclusion of only three databases in our search—with none from other disciplines like economics and international development or databases like Scopus and Web of Science—may have excluded relevant articles; however, we believe that this study achieved its function as a rapid review, synthesizing the current literature in a simplified and timely manner as suggested by the WHO framework for rapid reviews, [7] and can therefore serve as preliminary exploration of this specific topic, with future research targeting more comprehensive systematic reviews. Furthermore, the study only included articles that included at least one West African country which may limit the generalizability of the study findings. The *Hawker Tool* is most frequently used to assess risk of bias for qualitative studies; however, it was chosen for this study as the tool was designed with the idea that literature reviews are dynamic [8]. Thus, the tool addresses the challenges involved in identifying and assessing material involving a diversity of methodologies [8]. In addition, some might argue that the inclusion of commentaries in our search strategy and results leaves room for personal bias, however, given the complexity and importance of lived experiences surrounding the topic of the brain drain, it is pertinent that key voices are included. Despite these limitations, we believe that this review included relevant articles to understand the current literature on the medical brain drain, specifically from the critical region of West Africa to HICs.

## Conclusions

The brain drain has significant implications for global health equity, especially if the call to decolonize global health is to ever move from rhetoric to action. This rapid review aimed to understand the reasons for, implications of, and recommendations to mitigate physician emigration from West Africa. Overall, the reasons for physician emigration, as well as the implications on population health are well established in the literature. However, the literature is lacking in terms of effective solutions to manage physician emigration, particularly solutions that prioritize suggestions from voices within the continent. Multiple recommendations have been posed, however, it remains essential to prioritize collaborative efforts, at the health system and policy levels, between HICs and LMICs. Thus, future studies should focus on comprehensive systematic reviews that summarize effective, equity-based recommendations targeting physician emigration, as well as innovative interventions and policies to bring both supplier and receiver countries together to address physician emigration.

## Data Availability

All data generated or analyzed during this study are included in this published article and its supplementary information files.

## Abbreviations

ABCD: Asset-based community development
HICs: High-income countries
HRH: Human resources for health
LMICs: Low- and middle-income countries
MeSH: Medical Subject Headings
PRISMA: Preferred reporting items for systematic reviews and meta-analyses
WHO: World Health Organization
